# Combined Therapy with SS31 and Mitochondria Mitigates Myocardial Ischemia-Reperfusion Injury in Rats

**DOI:** 10.3390/ijms19092782

**Published:** 2018-09-15

**Authors:** Fan-Yen Lee, Pei-Lin Shao, Christopher Glenn Wallace, Sarah Chua, Pei-Hsun Sung, Sheung-Fat Ko, Han-Tan Chai, Sheng-Ying Chung, Kuan-Hung Chen, Hung-I Lu, Yi-Ling Chen, Tien-Hung Huang, Jiunn-Jye Sheu, Hon-Kan Yip

**Affiliations:** 1 Division of Thoracic and Cardiovascular Surgery, Department of Surgery, Kaohsiung Chang Gung Memorial Hospital and Chang Gung University College of Medicine, Kaohsiung 83301, Taiwan; fanyenlee2015@gmail.com (F.-Y.L.); luhung@adm.cgmh.org.tw (H.-I.L.); 2 Division of Cardiovascular Surgery, Department of Surgery, Tri-Service General Hospital, National Defense Medical Center, Taipei 11490, Taiwan; 3 Department of Nursing, Asia University, Taichung 41354, Taiwan; m8951016@gmail.com; 4 Department of Plastic Surgery, University Hospital of South Manchester, Manchester M23 9LT, UK; c.g.wallace@gmail.com; 5 Division of Cardiology, Department of Internal Medicine, Kaohsiung Chang Gung Memorial Hospital and Chang Gung University College of Medicine, Kaohsiung 83301, Taiwan; chuasr409@hotmail.com (S.C.); e12281@cgmh.org.tw (P.-H.S.); chaiht@mail.cgmh.org.tw (H.-T.C.); miosheny@gmail.com (S.-Y.C.); rylchen.msu@gmail.com (Y.-L.C.); tienhunghuang@gmail.com (T.-H.H.); 6 Department of Radiology, Kaohsiung Chang Gung Memorial Hospital and Chang Gung University College of Medicine, Kaohsiung 83301, Taiwan; sfatko@adm.cgmh.org.tw; 7 Department of Anesthesiology, Kaohsiung Chang Gung Memorial Hospital and Chang Gung University College of Medicine, Kaohsiung 83301, Taiwan; amigofx35@gmail.com; 8 Institute for Translational Research in Biomedicine, Kaohsiung Chang Gung Memorial Hospital, Kaohsiung 83301, Taiwan; 9 Center for Shockwave Medicine and Tissue Engineering, Kaohsiung Chang Gung Memorial Hospital and Chang Gung University College of Medicine, Kaohsiung 83301, Taiwan; 10 Department of Medical Research, China Medical University Hospital, China Medical University, Taichung 40402, Taiwan

**Keywords:** ischemia-reperfusion, oxidative stress, mitochondria, SS31, left ventricular ejection fraction

## Abstract

Myocardial ischemia-reperfusion (IR) injury contributes to adverse cardiac outcomes after myocardial ischemia, cardiac surgery, or circulatory arrest. In this study, we evaluated the ability of combined SS31-mitochondria (Mito) therapy to protect heart cells from myocardial IR injury. Adult male SD rats (*n* = 8/each group) were randomized: group 1 (sham-operated control), group 2 (IR, 30-min ischemia/72 h reperfusion), group 3 (IR-SS31 (2 mg intra-peritoneal injection at 30 min/24 h/48 h after IR)), group 4 (IR-mitochondria (2 mg/derived from donor liver/intra-venous administration/30 min after IR procedure)), and group 5 (IR-SS31-mitochondria). In H9C2 cells, SS31 suppressed menadione-induced oxidative-stress markers (NOX-1, NOX-2, oxidized protein) while it increased SIRT1/SIRT3 expression and ATP levels. In adult male rats 72 h after IR, left ventricular ejection fraction (LVEF) was highest in sham-operated control animals and lowest in the IR group. LVEF was also higher in IR rats treated with SS31-Mito than untreated IR rats or those treated with Mito or SS31 alone. Areas of fibrosis/collagen-deposition showed the opposite pattern. Likewise, levels of oxidative-stress markers (NOX-1, NOX-2, oxidized protein), inflammatory markers (MMP-9, CD11, IL-1β, TNF-α), apoptotic markers (mitochondrial-Bax, cleaved-caspase-3, PARP), fibrosis markers (p-Smad3, TGF-β), DNA-damage (γ-H2AX), sarcomere-length, and pressure/volume overload markers (BNP, β-MHC) all showed a pattern opposite that of LVEF. Conversely, anti-apoptotic (BMP-2, Smad1/5) and energy integrity (PGC-1α/mitochondrial cytochrome-C) markers exhibited a pattern identical to that of LVEF. This study demonstrates that the combined SS31-Mito therapy is superior to either therapy alone for protecting myocardium from IR injury and indicates that the responsible mechanisms involved increased SIRT1/SIRT3 expression, which suppresses inflammation and oxidative stress and protects mitochondrial integrity.

## 1. Introduction

Myocardial ischemia-reperfusion (IR), which is a pathological condition, is characterized by loss of the coronary blood supply to the myocardium followed by the restoration of perfusion as exemplified by acute myocardial infarction (AMI) during primary coronary intervention [1]. Myocardial IR injury contributes to adverse cardiac events after myocardial ischemia/AMI, cardiac surgery, cardiogenic shock, or circulatory arrest [2,3,4,5,6]. IR injury involves not only intracellular injury processes, generation of reactive oxygen species (ROS)/oxygen free radicals, but also injurious inflammatory and immune reactions [1,2,7,8,9,10,11,12]. The generated ROS interact with ion channels, sarco-endoplasmic reticulum, calcium-release channels, and myofilament proteins associated with excitation-contraction coupling, which influences intracellular ATP concentrations and pH [12,13]. Additionally, by impairing ATPase-dependent ion transport, ischemia increases intracellular and mitochondrial calcium levels (calcium overload) [12,13,14]. Moreover, ROS plays a crucial role to turn on the mitochondrial permeability transition pore, which results in the release of mitochondrial cytochrome C and other elements that further lead to cellular hyper-contracture and death [14,15]. Lastly, this leads to the loss of cardiac contractility and alterations in cardiovascular function [16,17]. Although there have been decades of extensive investigation regarding advanced pharmaco-therapeutic management and state-of-the-art methods for obstructive coronary artery interventions, efficacious therapies for myocardial IR injury remains lacking [12,13,14,15]. Accordingly, an alternative safe and efficacious treatment modality needs to be found.

SS31 is a potent antioxidant and is a cell-permeable peptide that resides in the inner mitochondrial membrane and possesses mitochondrial protective abilities [18,19,20,21]. SS31 can act as a scavenger of ROS/oxygen free radicals, can attenuate the generation of mitochondrial ROS, and can inhibit mitochondrial permeability transition [18,19,20,21,22]. In animal models of acute ischemia-reperfusion injury, SS31 has been shown to protect cells from apoptosis and necrosis induced by oxidative stress and inhibition of the mitochondrial electron transport chain [18,19,20,21].

Recent studies have demonstrated that mitochondrial transfusion can effectively protect against acute organ damage including acute lung injury [23,24] and acute ischemia-reperfusion injury of the heart [25] and liver [26]. The proposed mechanisms of mitochondrial transfusion protecting organs from IR injury consist of the inhibition of inflammation and oxidative stress as well as refreshment of mitochondria in the damaged cells [16,17,18,19,20,21,22,23]. Since many different deleterious events participate in IR, combination therapies are likely to be more effective than a monotherapy.

In this study, we evaluated the therapeutic potential of combined mitochondria replacement and SS31 treatment using a rodent model of acute myocardial IR injury. Additionally, to elucidate the underlying mechanisms of SS31 therapy for protecting cardiomyocytes, H9C2 cells were utilized for cell culturing a series of *in vitro* studies.

## 2. Results

### 2.1. SS31 Suppresses Menadione-Induced Oxidative Stress and Preserves ATP in H9C2 Cells

**Figure 1:**
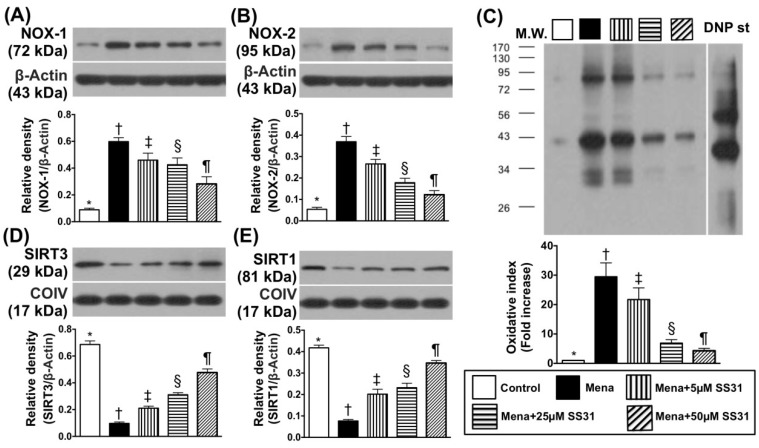
SS31 inhibits menadione-induced oxidative stress in H9C2 cells. (**A**) Protein expression of NOX-1 * vs. other groups with different symbols (†, ‡, §, ¶), *p* < 0.0001. (**B**) Protein expression of NOX-2 * vs. other groups with different symbols (†, ‡, §, ¶), *p* < 0.0001. (**C**) Oxidized protein expression * vs. other groups with different symbols (†, ‡, §, ¶), *p* < 0.0001. (Note: left and right lanes shown on the upper panel represent a protein molecular weight marker and control oxidized molecular protein standard, respectively). M.W = molecular weight. DNP = 1-3 dinitrophenylhydrazone. (**D**) Protein expression of mitochondrial SIRT3 * vs. other groups with different symbols (†, ‡, §, ¶), *p* < 0.0001. (**E**) Protein expression of mitochondrial SIRT1 * vs. other groups with different symbols (†, ‡, §, ¶), *p* < 0.0001. All statistical analyses were performed by one-way ANOVA and followed by the Bonferroni multiple comparison post hoc test (*n* = 6 for each group). Symbols (*, †, ‡, §, ¶) indicate significance at the 0.05 level.

First, we evaluated oxidative stress in menadione-treated H9C2 cells. Compared with the control group, protein expression of NADPH oxidase (NOX)-1, which is an indicator of oxidative stress, was increased in H9C2 cells treated with menadione (25 μM), which induces oxidative stress (Figure 1A). Importantly, the increased NOX-1 expression was dose-dependently suppressed by SS31 added into the cell culture media, which suggests that SS31 inhibits the oxidative stress (Figure 1A). Additionally, protein levels of NOX-2 (Figure 1B) and oxidized protein (Figure 1C), which are other indicators of oxidative stress, exhibited similar patterns as the NOX-1 expression.

Protein expressions of SIRT3 (Figure 1D), which is a member of the sirtuin family that is localized primarily to the mitochondria/nucleus, showed an opposite pattern to NOX-1/NOX-2. Furthermore, protein expression of SIRT1 (Figure 1E), which is another member of the sirtuin family that is localized primarily to the cytoplasm/nucleus also showed a similar pattern to SIRT3.

**Figure 2:**
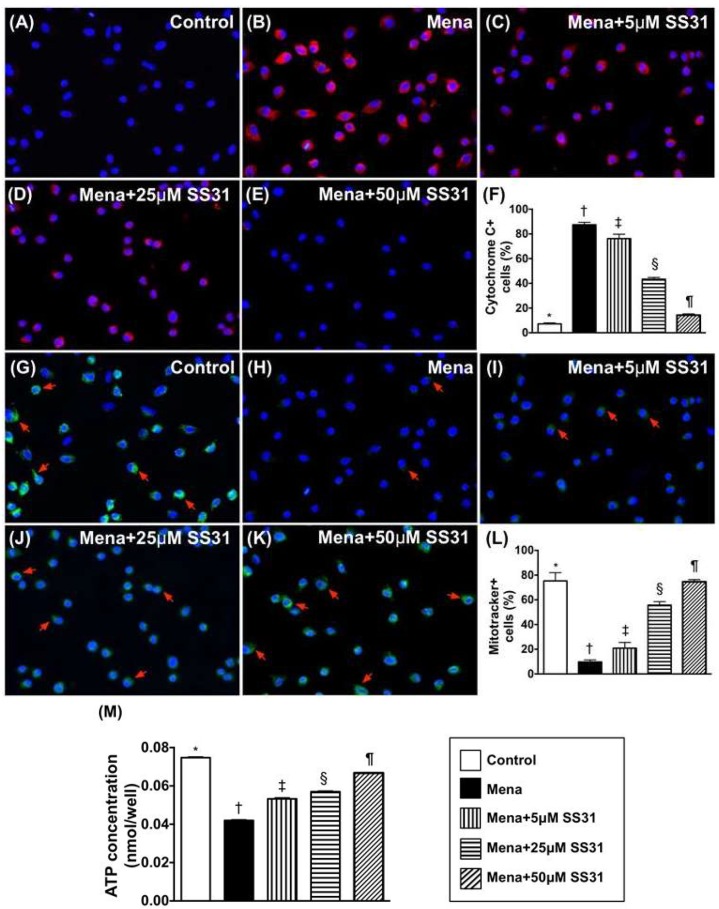
SS31 protects mitochondria from oxidative damage. (**A**–**E**) Illustrating the IF microscopic finding (400×) of cytosolic cytochrome C (cyt-Cyto C) staining (red color) (i.e., an indicator of mitochondrial damage). The ultrastructural cytosolic cytochrome C in H9C2 cells was notably increased by menadione treatment when compared to the normal control that was notably preserved after SS31 treatment. Blue color indicates nuclei stained by DAPI. (**F**) The analytical result of number of cyt-Cyto C+ cells * vs. other groups with different symbols (†, ‡, §, ¶), *p* < 0.0001. (**G**–**K**) Illustrating IF microscopic finding (400×) of mitotracker-positively stained cells (i.e., endogenous mitochondria) (green color) (red arrows). (**L**) The analytic result of number of mitotracker+ cells * vs. other groups with different symbols (†, ‡, §, ¶), *p* < 0.0001. (**M**) ATP concentration (i.e., measured by ELISA kit) of H9C2 cells * vs. other groups with different symbols (†, ‡, §, ¶), *p* < 0.0001. All statistical analyses were performed by one-way ANOVA and was followed by a Bonferroni multiple comparison post hoc test (*n* = 6 for each group). Symbols (*, †, ‡, §, ¶) indicate significance at the 0.05 level.

Immunofluorescence (IF) microscopy illustrated that the ultrastructural expression of cytosolic cytochrome C, which is an indicator of mitochondrial damage, was increased in menadione-treated (25 μM) H9C2 cells when compared to control cells (Figure 2A–F). Additionally, endogenous mitochondria (stained by mitotacker) were reduced in menadione-treated H9C2 cells compared to control cells (Figure 2G–L).

ATP concentration (Figure 2M) in H9C2 cells was identical to the expression of endogenous mitochondria in cells treated with menadione and SS31. These findings suggest that SS31 therapy preserves mitochondrial ATP levels in H9C2 cells by protecting mitochondria against oxidative-stress damage.

### 2.2. Mitochondrial Transfusion Refreshes Intracellular Mitochondria and SIRT3 Suppression Abrogates the Cyto-Protective Effects of SS31 by Increasing Oxidative Stress in Menadione-Treated H9C2 Cells

**Figure 3:**
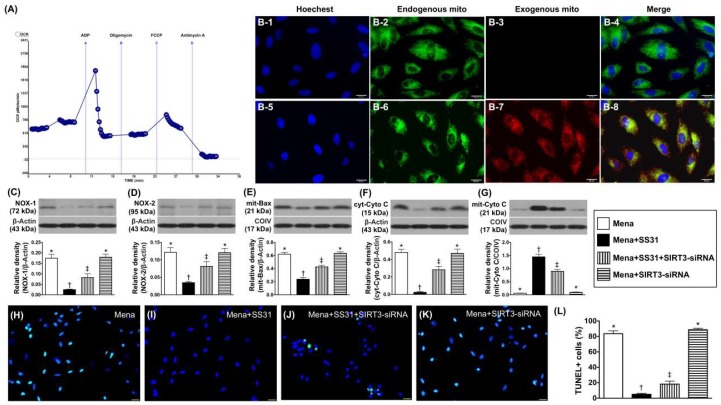
Refreshment of mitochondria in H9C2 cells and SS31 cyto-protective effect abolished by SIRT3 suppression. (**A**) Activity (OCR) of isolated mitochondria (determined by the Mito stress test kit and the XF^e^24 Analyzer) (*n* = 4). (**B**) Immunofluorescent (IF) (400×) staining showing nuclear DAPI stain (**B-1**,**B-5**), endogenous mitochondria (**B-2**,**B-6**), exogenous mitochondria (red color) being transferred into H9C2 cells (**B-7**), and (**B-3**) indicating that no exogenous mitochondria are transfused into H9C2 cells. (**B-4**) indicating merged of (**B-1**–**B-3**), and (**B-8**) indicating merged pictures of (**B-5**–**B-7**) with a yellow-green color indicating the fusion of exogenous and endogenous mitochondria in H9C2 cells. (**C**) Protein expression of NOX-1, * vs. other groups with different symbols (†, ‡), *p* < 0.0001. (**D**) Protein expression of NOX-2 * vs. other groups with different symbols (†, ‡), *p* < 0.0001. (**E**) Protein expression of mitochondrial Bax * vs. other groups with different symbols (†, ‡), *p* < 0.0001. (**F**) Protein expression cytosolic cytochrome C (cyt-Cyto C) * vs. other groups with different symbols (†, ‡), *p* < 0.0001. (**G**) Protein expression of mitochondrial cytochrome C (mit-Cyto C) * vs. other groups with different symbols (†, ‡), *p* < 0.0001. (**H**–**K**) Illustrating the microscopic finding (400×) of TUNEL assay for the identification of apoptotic nuclei (green color) in H9C2 cells. (**L**) Analytical results of the number of apoptotic nuclei * vs. other groups with different symbols (†, ‡), *p* < 0.0001. The blue color indicated nuclei stained by DAPI. All statistical analyses were performed by one-way ANOVA, which was followed by a Bonferroni multiple comparison post hoc test (*n* = 6 for each group). Symbols (*, †, ‡) indicate significance at the 0.05 level. Mena = menadione.

**Figure 4:**
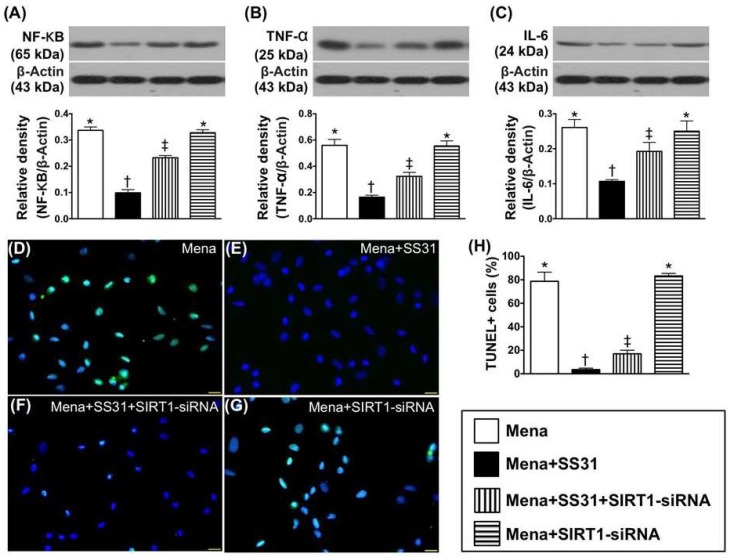
SIRT1 suppression inhibits SS31-mediated cyto-protective effect. (**A**) Protein expression of nuclear factor (NF)-κB, * vs. other groups with different symbols (†, ‡), *p* < 0.0001. (**B**) Protein expression of tumor necrosis factor (TNF)-α * vs. other groups with different symbols (†, ‡), *p* < 0.0001. (**C**) Protein expression of interleukin (IL)-6 * vs. other groups with different symbols (†, ‡), *p* < 0.0001. (**D**–**G**) Illustrating the immunofluorescent microscopic finding (400×) of TUNEL assay for identification of apoptotic nuclei (green color) in H9C2 cells (green color). (**H**) Analytical result of the number of apoptotic nuclei * vs. other groups with different symbols (†, ‡), *p* < 0.0001. All statistical analyses were performed by one-way ANOVA, which was followed by a Bonferroni multiple comparison post hoc test (*n* = 6 for each group). Symbols (*, †, ‡) indicate significance at the 0.05 level. Mena = menadione.

**Figure 5:**
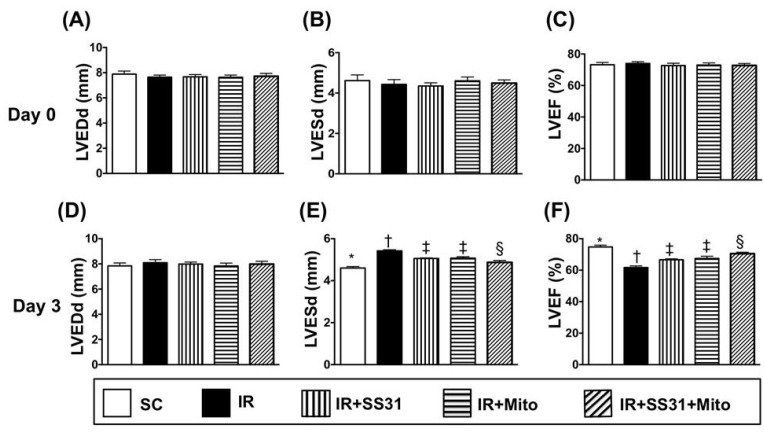
Transthoracic echocardiographic findings after the IR procedure. (**A**) At baseline, the left ventricular end-diastolic dimension (LVEDd), *p* > 0.5. (**B**) At baseline, left ventricular end-systolic dimension (LVESd), *p* > 0.5. (**C**) At baseline, left ventricular ejection fraction (LVEF), *p* > 0.5. (**D**) Upon 72 h after myocardial IR procedure, LVEDd, *p* > 0.3. (**E**) Upon 72 h after myocardial IR procedure, LVESd * vs. other groups with different symbols (†, ‡, §), *p* < 0.001. (**F**) Upon 72 h after myocardial IR procedure, LVEF, * vs. other groups with different symbols (†, ‡, §), *p* < 0.001. All statistical analyses were performed by one-way ANOVA, which was followed by a Bonferroni multiple comparison post hoc test (*n* = 8 for each group). Symbols (*, †, ‡, §) indicate significance at the 0.05 level. SC = sham control. IR = ischemia-reperfusion. Mito = mitochondria.

**Figure 6:**
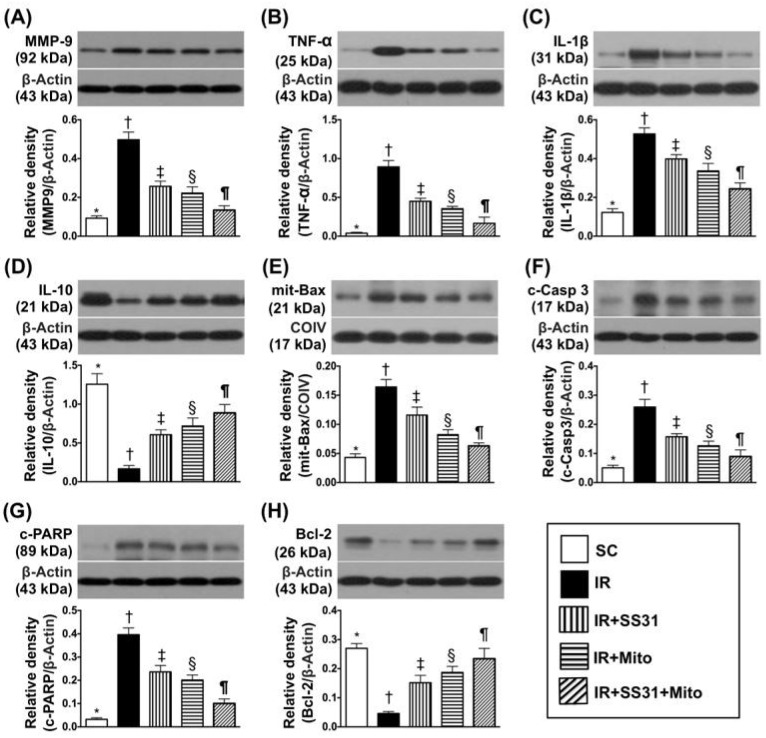
Pro-inflammatory, anti-inflammatory, apoptotic, and anti-apoptotic biomarkers in LV myocardium IR procedure. (**A**) Protein expression of matrix metalloproteinase (MMP)-9 * vs. other groups with different symbols (†, ‡, §, ¶), *p* < 0.0001. (**B**) Protein expression of tumor necrosis factor (TNF)-α * vs. other groups with different symbols (†, ‡, §, ¶), *p* < 0.0001. (**C**) Protein expression of interleukin (IL)-1ß * vs. other groups with different symbols (†, ‡, §, ¶), *p* < 0.0001. (**D**) Protein expression of IL-10 * vs. other groups with different symbols (†, ‡, §, ¶), *p* < 0.0001. (**E**) Protein expression of mitochondrial (mito)-Bax * vs. other groups with different symbols (†, ‡, §, ¶), *p* < 0.0001. (**F**) Protein expression of cleaved caspase 3 (c-Casp 3) * vs. other groups with different symbols (†, ‡, §, ¶), *p* < 0.0001. (**G**) Protein expression of cleaved poly (ADP-ribose) polymerase (c-PARP) * vs. other groups with different symbols (†, ‡, §, ¶), *p* < 0.0001. (**H**) Protein expression of Bcl-2 * vs. other groups with different symbols (†, ‡, §, ¶), *p* < 0.0001. All statistical analyses were performed by one-way ANOVA, which was followed by the Bonferroni multiple comparison post hoc test (*n* = 8 for each group). Symbols (*, †, ‡, §, ¶) indicate significance at the 0.05 level. SC = sham control, IR = ischemia-reperfusion, Mito = mitochondria.

**Figure 7:**
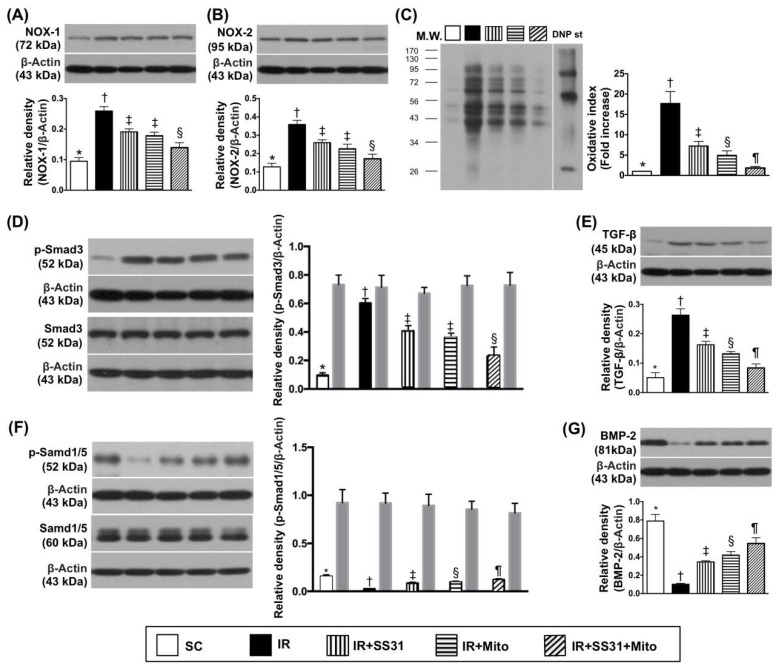
Oxidative-stress biomarkers in LV myocardium after the IR procedure. (**A**) Protein expression of NOX-1 * vs. other groups with different symbols (†, ‡, §), *p* < 0.0001. (**B**) Protein expression of NOX-2 * vs. other groups with different symbols (†, ‡, §), *p* < 0.0001. (**C**) Oxidized protein expression * vs. other groups with different symbols (†, ‡, §, ¶), *p* < 0.0001. (Note: left and right lanes shown on the upper panel represent protein molecular weight marker and control oxidized molecular protein standard, respectively). M.W = molecular weight. DNP = 1-3 dinitrophenylhydrazone. (**D**) Protein expression of phosphorylated (p)-Smad3 * vs. other groups with different symbols (†, ‡, §), *p* < 0.0001. Protein expression of total Smad3 (i.e., gray-colored bar chart) did not differ among the five groups (*p* > 0.5). (**E**) Protein expression of transforming the growth factor (TGF)-ß * vs. other groups with different symbols (†, ‡, §, ¶), *p* < 0.0001; (**F**) Protein expression of p-Smad1/5, * vs. other groups with different symbols (†, ‡, §, ¶), *p* < 0.0001. (**G**) Protein expression of bone morphogenetic protein (BMP-2) * vs. other groups with different symbols (†, ‡, §, ¶), *p* < 0.0001. All statistical analyses were performed by one-way ANOVA, which was followed by the Bonferroni multiple comparison post hoc test (*n* = 8 for each group). Symbols (*, †, ‡, §, ¶) indicate significance at the 0.05 level. SC = sham control, IR = ischemia-reperfusion, and Mito = mitochondria.

**Figure 8:**
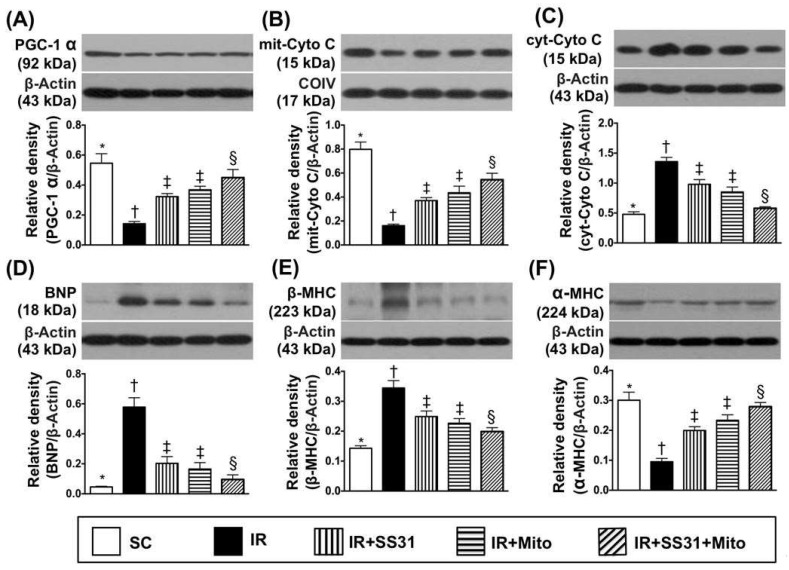
Energy integrity and pressure/volume overload biomarkers in LV myocardium after IR procedure. (**A**) Protein expression of Peroxisome proliferator-activated receptor gamma coactivator 1-alpha (PGC-1α) * vs. other groups with different symbols (†, ‡, §), *p* < 0.0001. (**B**) Protein expression of mitochondrial cytochrome C (mit-Cyto C) * vs. other groups with different symbols (†, ‡, §), *p* < 0.0001. (**C**) Protein expression of cytosolic cytochrome C (cyt-Cyto C) * vs. other groups with different symbols (†, ‡, §), *p* < 0.0001. (**D**) Protein expressions of brain natriuretic peptide (BNP) * vs. other groups with different symbols (†, ‡, §), *p* < 0.0001. (**E**) Protein expression of ß-myosin heavy chain (β-MHC) * vs. other groups with different symbols (†, ‡, §), *p* < 0.0001. (**F**) Protein expression of α-MHC * vs. other groups with different symbols (†, ‡, §), *p* < 0.0001. All statistical analyses were performed by one-way ANOVA and was followed by the Bonferroni multiple comparison post hoc test (*n* = 8 for each group). Symbols (*, †, ‡, §) indicate significance at the 0.05 level. SC = sham control, IR = ischemia-reperfusion, and Mito = mitochondria.

We used the Mito stress test kit and XF^e^24 Analyzer (seahorse method) to measure the oxygen consumption rate (OCR). The results showed high OCRs of isolated mitochondria, which indicates that the mitochondria isolation method was reliable (Figure 3A). IF microscopy demonstrated that numerous exogenous mitochondria were transfused into H9C2 cells (Figure 3(B-1–B-8)). This suggests that mitochondrial cell refreshment can be created by exogenous administration.

In vitro studies showed that the protein expression of NOX-1 was significantly lower in Mena + SS31 than in other groups, which is significantly lower in Mena + SIRT3-siRNA than in Mena only and Mena + SS31 + SIRT3-siRNA, but it showed no difference between the latter two groups (Figure 3C). Additionally, protein expression of NOX-2 displayed a similar pattern to the NOX-1 expression among the four groups (Figure 3D). Furthermore, protein expressions of mitochondrial Bax (an indicator of apoptosis) (Figure 3E) and cytosolic cytochrome C (an index of mitochondrial damage) (Figure 3F) exhibited an identical pattern while the protein expression of mitochondrial cytochrome C (an indicator of mitochondrial integrity) (Figure 3G) exhibited an opposite pattern to NOX-1 among the four groups. Moreover, the TUNEL assay showed that the apoptotic nuclei of H9C2 cells also displayed an identical pattern of NOX-1 among the four groups (Figure 3H–L).

When the above in vitro studies were performed with SIRT1-siRNA, protein levels of the inflammatory markers named interleukin (IL)-6, NF-κB, and tumor necrosis factor (TNF)-α as well as apoptotic nuclei displayed similar patterns to cells undergoing oxidative stress (Figure 4A–H).

### 2.3. Transthoracic Echocardiographic Findings after IR Procedure

The baseline parameters including left ventricular end-diastolic diameter (LVEDd), left ventricular end-systolic diameter (LVESd), and left ventricular ejection fraction (LVEF) did not differ among the five groups (Figure 5A–C). However, 72 h after the IR procedure, the LVEF was the highest in the sham control (SC) and the lowest in IR. It was also higher in IR-SS31-mitochondria (Mito) than in IR-SS31 and IR-Mito but is not different between the latter two groups (Figure 5F). On the other hand, LVESd exhibited an opposite pattern to LVEF (Figure 5E), which suggests that SS31-Mito therapy inhibits left ventricular (LV) remodeling. However, the LVEDd was similar among the five groups (Figure 5D).

### 2.4. Inflammatory and Apoptotic Markers in LV Myocardium after the IR Procedure

Protein expression of matrix metalloproteinase (MMP)-9, which is a marker of inflammation, was the lowest in SC, the highest in IR, significantly lower in IR-SS31-Mito than in IR-SS31 and IR-Mito, and significantly lower in IR-Mito than in IR-SS31 (Figure 6A). Additionally, the pro-inflammatory cytokines of TNF-α (Figure 6B) and IL-1ß (Figure 6C) exhibited similar patterns as MMP-9 among the five groups. In contrast, protein expression of the anti-inflammatory cytokine IL-10 (Figure 6D) showed a reverse pattern among the five groups.

Protein levels of the pro-apoptotic markers and mitochondrial Bax (Figure 6E) cleaved caspase-3 (Figure 6F) and cleaved poly(ADP-ribose) polymerase (PARP) (Figure 6G). It also exhibited identical patterns to the pro-inflammatory markers among the five groups. On the other hand, protein expression of Bcl-2 (Figure 6H), which is a pro-survival marker, displayed an opposite pattern to the pro-apoptotic markers among the five groups.

### 2.5. Oxidative Stress and Fibrosis Markers in LV Myocardium after the IR Procedure

The oxidized protein level was the lowest in SC, the highest in IR, significantly lower in IR-SS31-Mito than in IR-SS31 and IR-Mito, and significantly lower in IR-Mito than in IR-SS31 (Figure 7C). Protein levels of NOX-1 (Figure 7A) and NOX-2 (Figure 7B), which are another two indicators of oxidative stress, exhibited identical patterns as oxidized protein except for no difference between IR-SS31 and IR-Mito.

Protein expression of phosphorylated (p)-Smad3 (Figure 7D), which is an indicator of fibrosis, displayed a similar pattern to NOX-1 and NOX-2 among the five groups. Additionally, protein expression of transforming growth factor (TGF)-β (Figure 7E), which is another indicator of fibrosis, displayed an identical pattern as oxidative stress among the five groups. On the other hand, protein levels of p-Smad1/5 (Figure 7F) and bone morphogenetic protein (BMP)-2 (Figure 7G), which are two markers of anti-fibrosis, displayed an opposite pattern to oxidative stress among the five groups.

### 2.6. Energy Integrity and Pressure/Volume Overload Biomarkers in LV Myocardium after IR Procedure

Protein expression of peroxisome proliferator activated the receptor-gamma coactivator (PGC)-1α, which is a transcriptional coactivator that regulates genes involved in energy metabolism, was highest in SC, lowest in IR, and significantly higher in IR-SS31-Mito than in IR-SS31 and IR-Mito. However, it showed no difference between IR-Mito and IR-SS31 (Figure 8A). Additionally, protein expression of mitochondrial cytochrome C, which is an indicator of mitochondrial integrity, followed an identical pattern to PGC-α among the five groups (Figure 8B). On the other hand, protein expression of cytosolic cytochrome C, which is an indicator of mitochondrial damage, revealed an opposite pattern to PGC-α among the five groups (Figure 8C).

Protein levels of BNP (Figure 8D) and β-MHC (Figure 8E), which is an indicator of pressure overload, displayed an opposite manner to PGC-α among the five groups (Figure 8). On the other hand, protein expression of α-MHC (Figure 8F), which is a reversed myocardial hypertrophic marker, demonstrated a contrary pattern to β-MHC among the five groups.

### 2.7. Infarct and Collagen-Deposition Areas in LV Myocardium after the IR Procedure

**Figure 9:**
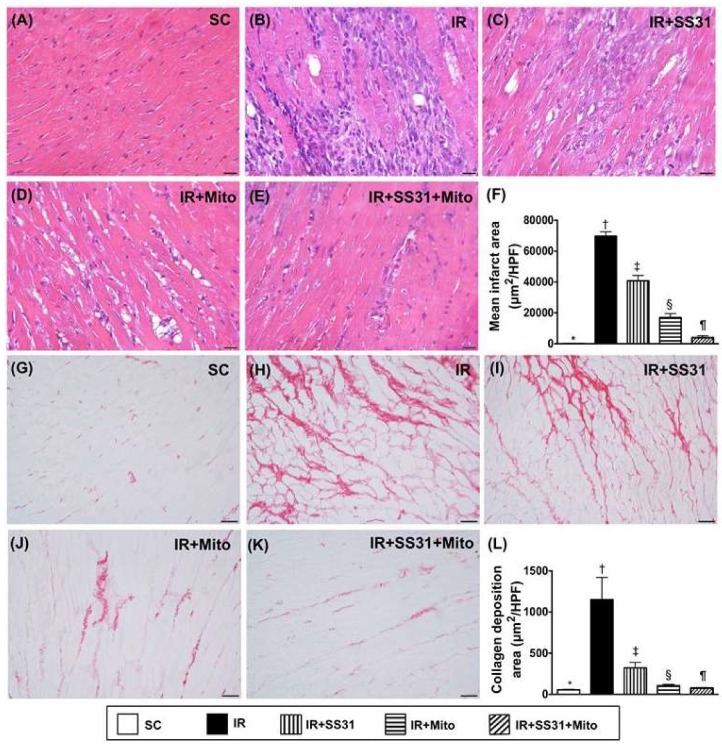
Infarct and collagen-deposition areas in the LV myocardium after the IR procedure. (**A**–**E**) Showing the light microscopic finding (400×) of H.E., stain in order to identify the infarct area (white-blue color). Scale bars in the right lower corner represents 20 µm. (**F**) Analytical result of the infarct area * vs. other groups with different symbols (†, ‡, §, ¶), *p* < 0.0001. (**G**–**K**) Illustrating the microscopic finding (200×) of Sirius red satin for the identification of collagen deposition area (pink color). (**L**) Analytical result of the collagen deposition area * vs. other groups with different symbols (†, ‡, §, ¶), *p* < 0.0001. Scale bars in the right lower corner represent 50 µm. All statistical analyses were performed by one-way ANOVA, which was followed by the Bonferroni multiple comparison post hoc test (*n* = 8 for each group). Symbols (*, †, ‡, §, ¶) indicate significance at the 0.05 level. SC = sham control, IR = ischemia-reperfusion, and Mito = mitochondria.

Light microscopy with H.E. staining showed that the infarct area was the lowest in SC, highest in IR, significantly lower in IR-SS31-Mito than in IR-SS31 and IR-Mito, and significantly lower in IR-Mito than in IR-SS31 (Figure 9A–F). Additionally, IHC microscopy showed that the collagen deposition area displayed an identical pattern of the infarct area among the five groups (Figure 9G–L).

### 2.8. Cellular Levels of Inflammatory Markers in LV Myocardium after IR Procedure

**Figure 10:**
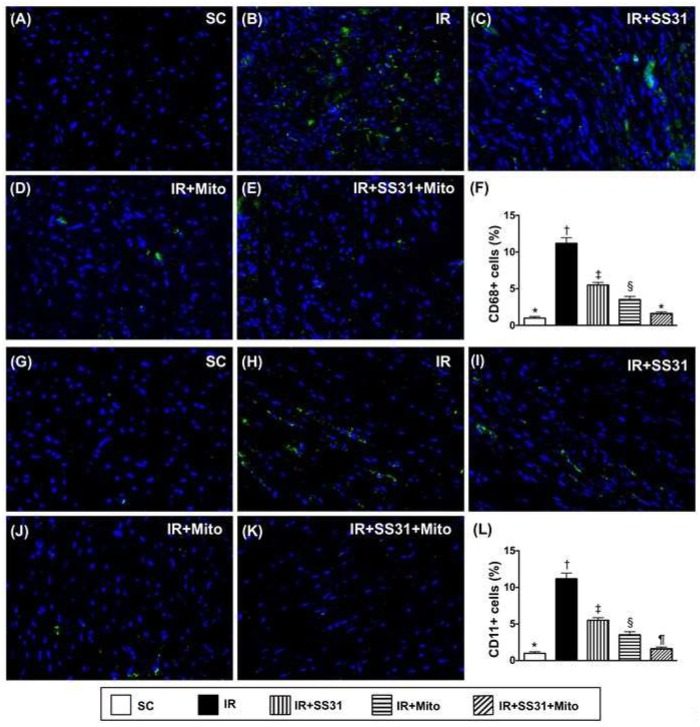
Inflammation in LV myocardium after the IR procedure. (**A**–**E**) Showing immunofluorescent (IF) microscopic finding (400×) of cellular expression of CD68 (green color). (**F**) Analytical result of number of CD68+ cells * vs. other groups with different symbols (†, ‡, §, ¶), *p* < 0.0001. (**G**–**K**) Illustration of IF microscopic finding (400×) of cellular expression of CD11 (green color). (**L**) Analytical result of number of CD11+ cells * vs. other groups with different symbols (†, ‡, §, ¶), *p* < 0.0001. Scale bars in the right lower corner represent 20 µm. All statistical analyses were performed by one-way ANOVA, which was followed by the Bonferroni multiple comparison post hoc test (*n* = 8 for each group). Symbols (*, †, ‡, §, ¶) indicate significance at the 0.05 level. SC = sham control, IR = ischemia-reperfusion, and Mito = mitochondria.

IF microscopy demonstrated that cellular expression of CD68, which is an indicator of inflammation, was the lowest in SC, the highest in IR, significantly lower in IR-SS31-Mito than in IR-SS31 and IR-Mito, and significantly lower in IR-Mito than in IR-SS31 (Figure 10A–F). Additionally, cellular expression of CD11, which is another indicator of inflammation, exhibited an identical pattern as CD68 among the five groups (Figure 10G–L).

### 2.9. Cellular Levels of DNA-Damage and Gap Junction Biomarkers in the LV Myocardium after the IR Procedure

**Figure 11:**
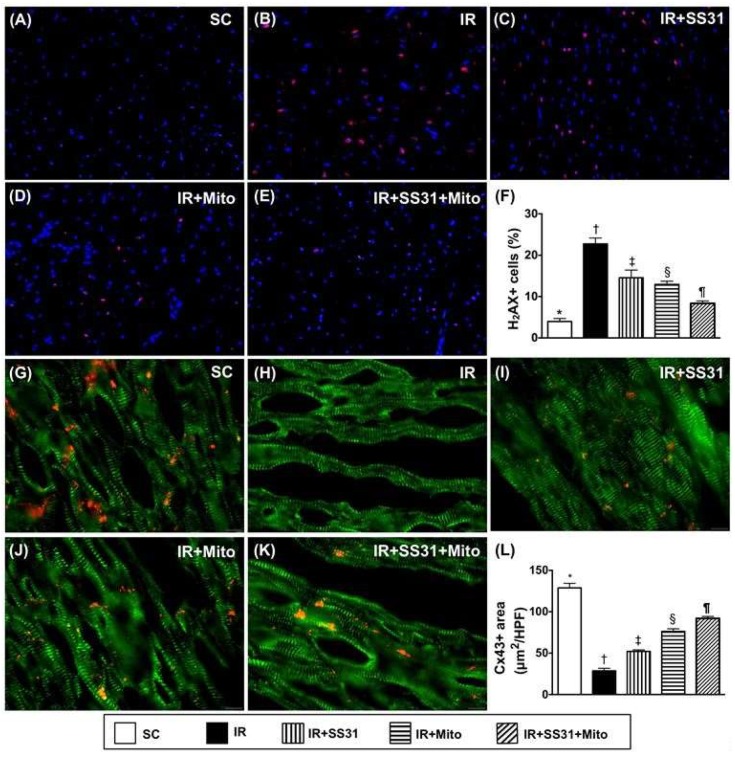
DNA-damage and Gap junction biomarkers in LV myocardium after the IR procedure. (**A**–**E**) Immunofluorescent (IF) microscopic finding (400×) of cellular expression of γ-H2AX (green). (**F**) Analytical result of number of γ-H2AX + cells * vs. other groups with different symbols (†, ‡, §, ¶), *p* < 0.0001. (**G**–**K**) IF microscopic finding (400×) of cellular expression of connexin43 (Cx43). (**L**) Analytical result of positively-stained Cx43 * vs. other groups with different symbols (†, ‡, §, ¶), *p* < 0.0001. Scale bars in the right lower corner represent 20 µm. All statistical analyses were performed by one-way ANOVA, which was followed by the Bonferroni multiple comparison post hoc test (*n* = 8 for each group). Symbols (*, †, ‡, §, ¶) indicate significance at the 0.05 level. SC = sham control. IR = ischemia-reperfusion. Mito = mitochondria.

IF microscopy demonstrated that the number of phosphorylated histone H2AX (γH2AX)^+^ cells, which was an indicator of DNA-damage, was the lowest in SC, the highest in IR, significantly lower in IR-SS31-Mito than in IR-SS31 and IR-Mito, and significantly lower in IR-Mito than in IR-SS31 (Figure 11A–F). On the other hand, the number of cells with positively stained connexin 43 (Cx43), which is a marker for the integrity of the gap junction for cell-to-cell communication, displayed an opposite pattern to γ-H2AX^+^ cells among the five groups (Figure 11G–L).

### 2.10. Sarcomere Length of LV Myocardium after the IR Procedure

**Figure 12:**
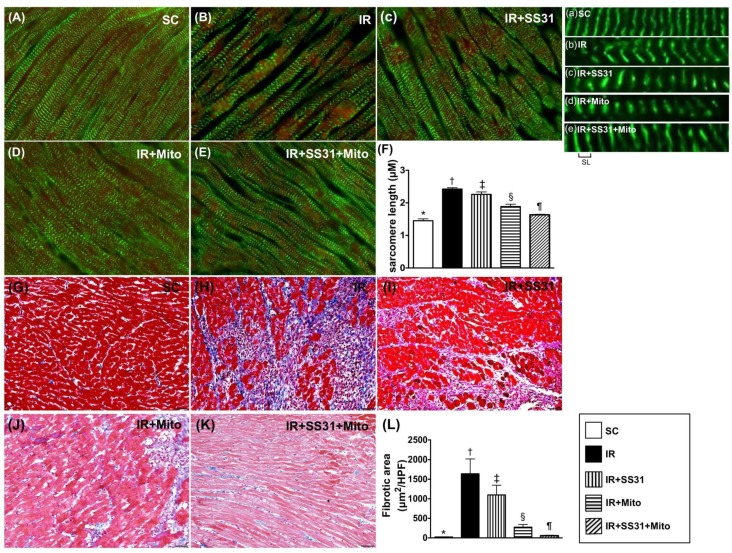
Actin-phalloidin staining (**A**–**E**) for measuring the sarcomere length of cardiomyocytes in LV myocardium after the IR procedure. (**A**–**E**) Immuno-fluorescent microscopic (1000×) finding for identifying the sarcomere length. (**a**–**e**) Illustrating the manifestations of distance of sarcomere length (SL) in five groups (i.e., (**A**–**E**)). (**F**) Results of statistical analysis of the changes in length of sarcomere identified by troponin-I staining * vs. other groups with different symbols (†, ‡, §, ¶), *p* < 0.0001. The scale bars in the right lower corner represent 10 µm. (**G**–**K**) Illustrating the Masson’s trichrome stain (200×) for identification of the fibrotic area (blue color). (**L**) Results of statistical analysis of the fibrotic area * vs. other groups with different symbols (†, ‡, §, ¶), *p* < 0.0001. All statistical analyses were performed by one-way ANOVA, which was followed by the Bonferroni multiple comparison post hoc test (*n* = 8 for each group). Symbols (*, †, ‡, §, ¶) indicate significance at the 0.05 level. SC = sham control, IR = ischemia-reperfusion, and Mito = mitochondria.

IF microscopy showed that the sarcomere length of LV myocardium was the lowest in SC, the highest in IR, significantly lower in IR-SS31-Mito than in IR-SS31 and IR-Mito, and significantly lower in IR-Mito than in IR-SS31. These results suggest that LV remodeling is initiated in response to myocardial IR injury that is suppressed by SS31-Mito treatment (Figure 12A–F). Additionally, the IHC microscopic finding of Masson’s trichrome stain showed that the fibrotic area displayed an identical pattern to sarcomere length among the five groups (Figure 12G–L).

### 2.11. The Expression of Mitochondrial DNA Copy Number in the IR Area of the Left Ventricular Myocardium

**Figure 13:**
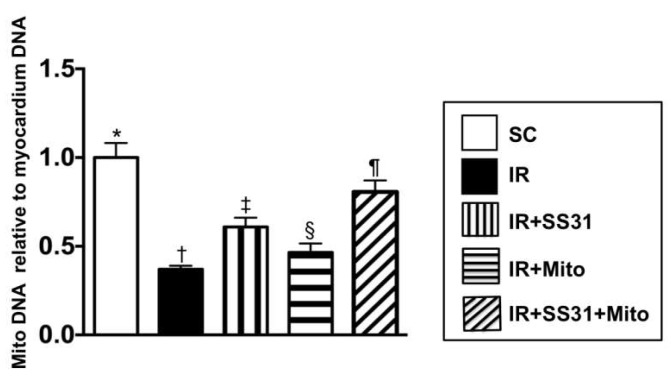
The relative mitochondrial DNA copy number to the left ventricular myocardium DNA copy number in the IR area Analytical result of mitochondrial DNA copy * vs. other groups with different symbols (†, ‡, §, ¶), *p* < 0.0001. All statistical analyses were performed by one-way ANOVA and followed by the Bonferroni multiple comparison post hoc test (*n* = 8 for each group). Symbols (*, †, ‡, §, ¶) indicate significance at the 0.05 level. SC = sham control, IR = ischemia-reperfusion, and Mito = mitochondria.

The qPCR demonstrated that the mitochondrial DNA copy number was the highest in SC, the lowest in IR, significantly higher in IR-SS31-Mito than in IR-SS31 and IR-Mito, and significantly lower in IR-Mito than in IR-SS31. This suggests that exogenous mitochondrial transfusion would increase mitochondrial content in the IR myocardium and would be further enhanced after SS31-Mito treatment (Figure 13).

## 3. Discussion

This study investigated the effectiveness of the combination SS31-mitochondria (Mito) treatment on protecting LV myocardium against the IR injury in rats. Myocardial IR injury rapidly elicited molecular-cellular perturbations in the LV myocardium, deteriorated the LV function, and induced LV remodeling. Mito therapy had comparable effects on preserving LV function as SS31 therapy, which reduced the molecular-cellular perturbations and LV remodeling after rodent myocardial IR injury. Importantly, combined SS31-Mito therapy further improved short-term outcomes after IR injury.

Previous studies have shown that mitochondria-targeted peptide therapy can protect organs from IR injury [20,27,28,29]. The most important finding in the present study was that, compared with SC, LVEF (i.e., functional aspect) was significantly reduced in IR animals. However, LVEF was preserved in IR animals after receiving SS31 or Mito treatment and was further improved after the combined SS31-Mito treatment. The transthoracic echocardiogram findings demonstrated that both LVEDs and LVESd (i.e., anatomical aspects) were increased in IR animals when compared to SC controls. This was reversed by SS31 or Mito treatment and was further reversed by the combined SS31-Mito therapy. Thus, our findings are in agreement with previous studies [20,27,28,29] and demonstrate that SS31-Mito therapy rescues LV dysfunction and abrogates LV remodeling after myocardial ischemia/infarction.

The infarct, fibrotic, and collagen deposition areas (i.e., histopathological assessment) were considerably higher in IR animals that in SC controls. These findings may explain why LVEF was reduced in IR animals. These three histopathological parameters were reduced in IR animals after SS31 or Mito treatment and further decreased after the combined SS31-Mito therapy. These findings could explain why the LVEF was significantly preserved in those of IR animals treated by SS31-Mito than in those of IR-only animals. Previous studies have revealed that myocardial IR injury contributes to the reduction of LV function and adverse cardiac events [2,3,4,5,6] especially in the setting of AMI. Our findings, therefore, suggest that mitochondria-targeted therapies may be an assistant option in clinical settings of IR injury especially in AMI patients with severe LV dysfunction.

Our results demonstrate that, compared with the control SC group, the IR animals exhibited increased inflammatory, oxidative stress, apoptotic, fibrotic, and DNA-damage biomarkers in LV myocardium while they had decreased levels of energy biogenesis (PGC-1α) and mitochondrial integrity (mitochondrial cytochrome C) biomarkers. These findings may explain why LVEF was lower in IR animals than in the SC control group. Importantly, these molecular-cellular perturbations were reversed in IR animals after receiving the SS31-Mito combination therapy.

Our previous studies have indicated an association between pressure/volume overload and upregulation of heart failure biomarkers [30,31,32,33]. In this study, we found that, compared to the control SC group, the IR animals exhibited increased heart failure biomarkers (BNP, ß-MHC) and sarcomere length while they had decreased levels of myocardial integrity biomarkers (connexin43, mitochondrial cytochrome-C, α-MHC, BMP-2, Smad1/5). Thus, our present findings reinforce our previous studies [30,31,32,33] and explain why LV remodeling (abnormal increase in LVEDd and LVESd) was augmented in IR animals. Importantly, these molecular-cellular perturbations were reversed and LVEF was preserved in IR animals after receiving the SS31-Mito combination treatment.

However, the exact underlying mechanisms leading to improvement of LV function in IR animals after receiving the SS31-Mito combination therapy remain unknown. A previous study has suggested that SIRT1 protects organs from IR injury by inhibiting the NF-κB activation, which results in the downregulation of inflammation [34]. However, other studies have indicated that SIRT3 protects organs from IR injury by preserving the mitochondrial functional integrity and by upregulating mitochondrial fusion and limiting mitochondrial fission, segregation, and depolarization [35], which inhibits the oxidative stress. Our in vitro data have shown that SS31 upregulates protein levels of SIRT1 and SIRT3 as well as ATP concentration and mitochondrial integrity and inhibits NF-κB and oxidative stress in H9C2 cells while SIRT1 or SIRT3 suppression has the opposite effects. These findings suggest a protective role of SIRT1/SIRT3 in IR organ injury and identify them as possible targets when improving heart function in IR animals. Additionally, our *in vitro* data indicate that the refreshment of endogenic mitochondria by exogenic mitochondria may explain the additional benefit of Mito therapy in protecting heart function in IR animals. Since the combined SS31-Mito therapy was superior in preserving heart function when compared to either therapy alone, the data indicate that the exogenous Mito may protect the heart cells from oxidative-stress damage.

Surprisingly, although the effect of exogenous mitochondrial therapy for protecting the myocardium against IR injury was established, the labeled exogenous mitochondria was not identified by IF microscopy in the heart specimen. Interestingly, our previous study [26] has also demonstrated only transient existence of exogenous mitochondria in liver in the rat model of liver IR injury undergoing the exogenous mitochondrial therapy. Our finding was comparable with the finding of our previous study [26].

**Figure 14:**
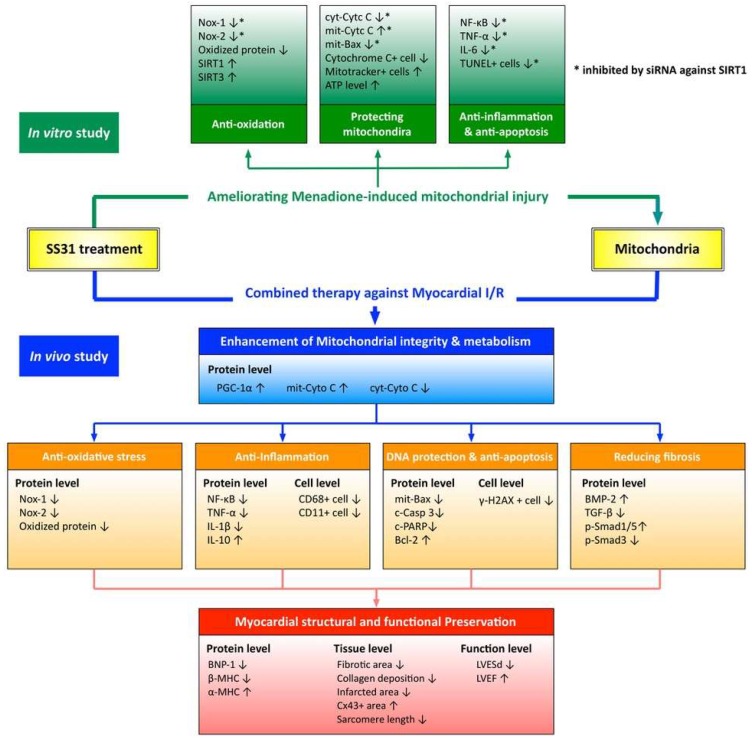
Proposed mechanisms underlying the positive therapeutic effects of combined SS31 and mitochondria preserved heart function in a setting of ischemia-reperfusion myocardial injury. The protective effects of combined SS31 and mitochondria on myocardium and heart function are completed by inhibiting inflammation, oxidative stress, cellular apoptosis, DNA/mitochondrial damage, and myocardial fibrosis.

Although extended works were doing very well in the present study, we only provided new direction rather than brand new findings. Future studies should elucidate the precise mechanisms of how the SS31-Mito combination therapy improves the heart function in an IR myocardial injury. The proposed mechanisms are summarized in Figure 14. In addition, since our study included only a 72-h follow-up period, future studies should analyze long-term outcomes.

## 4. Materials and Methods

### 4.1. Animal Studies

The protocols and procedures for animal study were approved by our Institute of Animal Care and Use Committee (Affidavit of Approval of Animal Use Protocol No. 2015061501 and the approval day was 1 July, 2015) and conducted in accordance with the Guide for the Care and Use of Laboratory Animals (The Eighth Edition of the Guide for the Care and Use of Laboratory Animals (NRC 2011)). Animals were housed in the AAALAC-approved animal facility in our institute with confined temperatures and light cycles (24 °C and 12/12 light cycles).

### 4.2. Induction of Acute Myocardial Ischemia-Reperfusion Injury

Pathogen-free, adult male Sprague-Dawley (SD) rats (*n* = 40) weighing 320–350 g (Charles River Technology, BioLASCO, Taipei, Taiwan) were divided into five groups: Sham control (SC) group, acute myocardial IR injury group, IR + SS31 (2 mg intra-peritoneal injection at 30 min/24 h/48 h after IR) group, IR + mitochondria group (Mito) ((2000 µg)/derived from the donor liver via intra-venous administration 30 min after the IR procedure), and the combination IR + SS31 + Mito group.

The procedure and protocol for myocardial IR injury were based on our recent report [36]. All animals were placed under anesthesia with 2.0% inhalational isoflurane on a warming pad at 37 °C for the IR procedure. Under sterile conditions, the heart was exposed via a left thoracotomy. IR injury was induced by ligating flow within the left coronary artery for 40 min with a 7-0 prolene suture and 3 mm distal to the margin of the left atrium. Myocardial ischemia was confirmed by identifying a myocardial color change from red to dark over the of left ventricular (LV) anterior wall along with quickly developing akinesia and dilatation. Animals that received thoracotomy only without IR induction served as sham-operated controls. The tight was then removed after 40-min ischemia, which was followed by 72 h reperfusion. The rats were sacrificed 72 h after the IR procedure and the hearts were harvested. The dosages of SS31 and mitochondria used were based on our recent reports [21,24,37].

### 4.3. Functional Assessment with Echocardiography

The procedure and protocol for echocardiography have been described in our recent report [30]. 2-D echocardiography was carried out in each animal before to and at day-3 after conduction of myocardial IR. An ultrasound machine (Vevo 2100, Visualsonics, New York, NY, USA). LV end-systolic diameter (ESD) and end-diastolic diameter (EDD) were examined at the mitral valve and LV papillary levels. The LVEF was calculated as follows: LVEF (%) = [(LVEDD^3^ − LVEDS^3^)/LVEDD^3^] × 100%.

### 4.4. Mitochondrial Isolation, Staining, and Transfusion

Mitochondrial isolation and mitotracker staining have been described in our recent report [38]. Briefly, liver mitochondria were isolated from six donor rats. The rats were starved overnight prior to the mitochondrial isolation procedure. The rats were then sacrificed and the livers were harvested. Immediately, each liver (3 g) was immersed in 50 mL of ice-cold IBc (10 mM Tris–MOPS, 5 mM EGTA/Tris and 200 mM sucrose, pH 7.4) and rinsed. The liver was then minced using scissors in a beaker on ice. IBc were discarded during the mincing and replaced with 18 mL of ice-cold fresh IBc. The liver was homogenized using a Teflon pestle. The homogenates were transferred to a 50 mL tube and centrifuged at 600× *g* for 10 min at 4 °C. The supernatants were transferred to centrifuge tubes for centrifugation at 7000× *g* for 10 min at 4 °C. The supernatants were discarded and the pellets were washed with 5 mL of ice-cold IBc. Again, the supernatants from pellets were centrifuged at 7000× *g* for 10 min at 4 °C. The supernatants were discarded and the pellets, which contained the mitochondria, were re-suspended. Concentration of the mitochondrial suspension was measured using the Biuret method. Each 10 mg of isolated mitochondria were labeled with 1 M of MitoTracker Red CMXRos (Invitrogen, Carlsbad, CA, USA) by incubating at 37 °C for 30 min.

The mitochondrial transfusion has been described in our recent report [38]. Briefly, intra-venous administration of mitochondria (2000 µg/each animal) at 30 min after IR was performed immediately after labeling (i.e., <2 h after the isolation procedure).

### 4.5. Oxygen Consumption Rate (OCR) of the Isolated Mitochondria (Seahorse Method)

Activity of isolated mitochondria from rat liver was determined by an Extracellular Flux Analyzer (XF^e^24, Seahorse Bioscience, North Billerica, MA, USA) by assessing the degree of coupling between the electron transport chain (ETC) and the oxidative phosphorylation machinery (OXPHOS), which was described in Reference [34]. Isolated mitochondria (10 µg/well) from rat liver were diluted in ice-cold 1X mitochondria assay solution (MAS) (70 mM sucrose, 220 mM mannitol, 10 mM KH_2_PO_4_, 5 mM MgCl_2_, 2 mM HEPES, 1.0 mM EGTA, pH 7.2) and centrifuged at 3000× *g* for 30 min. After attachment of mitochondria to the XF24 plate, the coupling reaction was initiated with the administration of substrate (10.0 mM succinate). State 3 was initiated with ADP (0.5 mM) while state 4 was induced with the addition of oligomycin (2 µM). Maximal uncoupler-stimulated respiration was elicited with FCCP (4 µM) while complex III repression was induced by antimycin A (4 µM). Lastly, OCR of isolated mitochondria was measured (Figure 3A).

### 4.6. Material and Method for In Vitro Study

To assess whether SS31 therapy could protect the cardiomyocytes against Mena (i.e., an indicator of oxidative damage) damage, the H9C2 cell line was replaced for the primary cardiomyocyte culture. Additionally, the experimental methods such as the seahorse method, the Western blot, ELISA, and Immunohistochemical (IHC) and immunofluorescent (IF) staining were utilized in the present study.

### 4.7. ATP Assay by ELISA

The ATP assay protocol was based on the manufacturer’s instructions. In detail, after removing H9C2 cells from culture plates, 100 μL of ATP Assay Buffer was added to the cells and was followed by centrifugation at 12,000 rpm at 4 °C for 5 min. Supernatants were transferred into micro-tubes and 10 µL of neutralization solution (ab204708, Abcam, Cambridge, MA, USA) was added and allowed to rest for 5 min on ice. Absorbance at 570 nm was measured in a dark room 30 min later.

### 4.8. Assessment of SS31 Role in Protecting Cardiomyocytes from Oxidative Stress 

To evaluate whether SS31 protects cardiomyocytes against acute oxidative stress injury, H9C2 cells were transfected with siRNA and treated with menadione (20 μM) with and without SS31 (50 μM, Figure 3). The dosage of menadione was based on our previous report with some modification [24].

### 4.9. Western Blot Analysis of Heart Tissues

Western blot analysis was performed as described previously [24,30,36,37,38]. In detail, equal amounts (50 μg) of protein extracts were loaded and separated by SDS-PAGE. Separated proteins were transferred to PVDF membranes and nonspecific sites were blocked by incubation in blocking buffer (5% nonfat dry milk in T-TBS (TBS containing 0.05% Tween 20)) overnight. The membranes were incubated with the indicated primary antibodies mitochondrial Bax (1:1000, Abcam), cleaved poly (ADP-ribose) polymerase (PARP) (1:1000, Cell Signaling, Beverly, MA, USA), caspase 3 (1:1000, Cell Signaling), Bcl-2 (1:200, Abcam), tumor necrotic factor (TNF)-α (1:1000, Cell Signaling), MMP-9 (1:3000, Abcam), interleukin (IL)-1β (1:1000, Cell Signaling), nuclear factor (NF)-κB (p65) (1:1000, Abcam), IL-6 (1:750, Abcam), NADPH oxidase (NOX)-1 (1:1500, Sigma, St. Louis, MO, USA), NOX-2 (1:500, Sigma), cytosolic cytochrome C (1:2000, BD, Franklin Lakes, NJ, USA), mitochondrial cytochrome C (1:2000, BD), peroxisome proliferator-activated receptor gamma coactivator 1-alpha (PGC-1α), transforming growth factor (TGF)-β (1:5000, Abcam), p-Smad1/5 (1:1000, Cell Signaling), bone morphogenetic protein (BMP)-2 (1:500, Abcam), brain natriuretic peptide (BNP) (1:500, Abcam), ß-myosin heavy chain (β-MHC) (1:300, Santa Cruz, Dallas, TX, USA), α-MHC (1:1000, Santa Cruz), SIRT1 (1:4000, Abcam), and SIRT3 (1:500, Abcam) for 1 h at room temperature. Horseradish peroxidase-conjugated anti-rabbit IgG (1:2000, Cell Signaling) was used as a secondary antibody. Immuno-reactive bands were visualized by enhanced chemiluminescence (ECL; Amersham Biosciences, Waltham, MA, USA) and digitized using Labwork software (UVP).

### 4.11. Immunohistochemical (IHC) and Immunofluorescent (IF) Staining

Re-hydrated paraffin sections were treated with 3% H_2_O_2_ for 30 min and incubated with an Immuno-Block reagent (BioSB, Santa Barbara, CA, USA) for 30 min at room temperature. Sections were then incubated with primary antibodies against matrix metalloproteinase (MMP)-9 (1:100, Abcam), CD11 (1:200, Abcam), γ-H2AX (1:500, Abcam), connexin43 (Cx43) (1:200, Merck Millipore, Temecula, CA, USA), troponin for sarcomere length (1:500, Bioss, Woburn, MA, USA), and cytochrome C (1:250, Abcam). Three sections of kidney specimens from each rat were analyzed. For quantification, three randomly selected HPFs (200× or 400× for IHC and IF studies) were analyzed in each section. The mean number of positively-stained cells per HPF for each animal was determined by summation of all numbers divided by 9. The distance between two sarcomeres, which indicated a sarcomere length, was measured by the IF microscope.

### 4.10. Oxidative Stress Reaction in the LV Myocardium

The Oxyblot Oxidized Protein Detection Kit was purchased from Chemicon (S7150). Expression of oxidative stress proteins has been described previously [24,30,36,37,38]. Immuno-reactive bands were visualized by ECL (Amersham Biosciences) and digitized using Labwork software (UVP).

### 4.12. Histological Quantification of Myocardial Fibrosis/Infarct Area (IA) and Collagen Deposition Area

This procedure and protocol were detailed in our previous report [36,39]. A total of 3 sections of LV myocardium in each group of animals were well-prepared at 4 µm thickness by Cryostat (Leica CM3050S). The integrated area (µm^2^) of IA and fibrotic area on each section were assessed using the Image Tool 3 (IT3) image analysis software (University of Texas, Health Science Center, San Antonio, TX, UTHSCSA, Image Tool for Windows, Version 3.0, USA). Additionally, 3 randomly selected HPFs were analyzed in each section. The mean pixel number per HPF in each animal was then assessed by the summation of all pixel numbers and was divided by 9. The mean LV fibrotic integrated area (µm^2^) per HPF was gained using a conversion factor of 19.24 (since 1 µm^2^ represented 19.24 pixels). Additionally, HPF (×100) of each section were utilized for the identification of Sirius red-positively stained areas in each section.

### 4.13. Quantification of Mitochondrial DNA Copy Number by qPCR

Total DNA was extracted by using the DNeasy Blood & tissue kit (Qiagen, Germantown, MD, USA) based on the manufacturer’s instructions. For assessment of the copy number of mitochondrial DNA, mitochondrial DNA (ND1-mtDNA, mitochondria specific DNA) was quantified by QuantiNOVA SYBR Green PCR assay (Qiagen) and normalized by rat genomic DNA (GAPDH-DNA, intronic DNA). Triplicate assays were performed for each sample by the Step One-Plus machine (ABI, Waltham, MA, USA). Primer sequences were listed below:

ND1-mtDNA forward: 5′-CTCCCTATTCGGAGCCCTAC-3′

ND1-mtDNA reverse: 5′-ATTTGTTTCTGCTAGGGTTG-3′

GAPDH-DNA forward: 5′-TAGGGCTGGAAAATCACTGG-3′

GAPDH-DNA reverse: 5′-GTATTCATCACCCCCACCAC-3′

### 4.14. Statistical Analysis

Quantitative data are expressed as means ± SD. Statistical analysis was performed by ANOVA and was followed by the Bonferroni multiple-comparison post hoc test. SAS statistical software for Windows version 8.2 (SAS institute, Cary, NC, USA) was utilized. A probability value <0.05 was considered statistically significant.

### 4.15. Availability of Data and Material

The datasets used and/or analyzed during the current study are available from the corresponding author on reasonable request.

## 5. Conclusions

In conclusion, we demonstrate that the combined SS31-Mito therapy is superior to either therapy alone for improving LV function and inhibiting LV remodeling in the setting of myocardial IR injury. The responsible mechanisms may involve the increased expression of SIRT1 and/or SIRT3, which suppresses inflammation and oxidative stress and protects mitochondrial integrity.

## Abbreviations

AMI	acute myocardial infarction
BMP	bone morphogenetic protein
BNP	brain natriuretic peptide
Casp	caspase
c	cleaved
Cx43	connexin 43
IL	interleukin
IR	ischemia-reperfusion
LVEF	left ventricular ejection fraction
LVEDd	left ventricular end-diastolic diameter
LVESd	left ventricular end-systolic diameter
MMP	matrix metalloproteinase
Mito	mitochondria
MHC	myosin heavy chain
NOX	NADPH oxidase
NF-κB	nuclear factor-κB
OCR	oxygen consumption rate
PGC	peroxisome proliferator activated receptor-gamma coactivator
PARP	poly(ADP-ribose) polymerase
ROS	reactive oxygen species
TGF	transforming growth factor
TNF-α	tumor necrosis factor-α

## References

[1] Eltzschig H.K., Eckle T. (2011). Ischemia and reperfusion—From mechanism to translation. Nat. Med..

[2] Chen Y.S., Chao A., Yu H.Y., Ko W.J., Wu I.H., Chen R.J., Huang S.C., Lin F.Y., Wang S.S. (2003). Analysis and results of prolonged resuscitation in cardiac arrest patients rescued by extracorporeal membrane oxygenation. J. Am. Coll. Cardiol..

[3] Ito H., Maruyama A., Iwakura K., Takiuchi S., Masuyama T., Hori M., Higashino Y., Fujii K., Minamino T. (1996). Clinical implications of the ‘no reflow’ phenomenon. A predictor of complications and left ventricular remodeling in reperfused anterior wall myocardial infarction. Circulation.

[4] Kloner R.A., Ellis S.G., Lange R., Braunwald E. (1983). Studies of experimental coronary artery reperfusion. Effects on infarct size, myocardial function, biochemistry, ultrastructure and microvascular damage. Circulation.

[5] Rokos I.C., French W.J., Koenig W.J., Stratton S.J., Nighswonger B., Strunk B., Jewell J., Mahmud E., Dunford J.V., Hokanson J. (2009). Integration of pre-hospital electrocardiograms and ST-elevation myocardial infarction receiving center (SRC) networks: Impact on Door-to-Balloon times across 10 independent regions. JACC Cardiovasc. Interv..

[6] Rokos I.C., Larson D.M., Henry T.D., Koenig W.J., Eckstein M., French W.J., Granger C.B., Roe M.T. (2006). Rationale for establishing regional ST-elevation myocardial infarction receiving center (SRC) networks. Am. Heart J..

[7] De Scheerder I., Vandekerckhove J., Robbrecht J., Algoed L., De Buyzere M., De Langhe J., De Schrijver G., Clement D. (1985). Post-cardiac injury syndrome and an increased humoral immune response against the major contractile proteins (actin and myosin). Am. J. Cardiol..

[8] Frangogiannis N.G. (2008). The immune system and cardiac repair. Pharmacol. Res..

[9] Frangogiannis N.G., Smith C.W., Entman M.L. (2002). The inflammatory response in myocardial infarction. Cardiovasc. Res..

[10] Lambert J.M., Lopez E.F., Lindsey M.L. (2008). Macrophage roles following myocardial infarction. Int. J. Cardiol..

[11] Lange L.G., Schreiner G.F. (1994). Immune mechanisms of cardiac disease. N. Engl. J. Med..

[12] Hoffman J.W., Gilbert T.B., Poston R.S., Silldorff E.P. (2004). Myocardial reperfusion injury: Etiology, mechanisms, and therapies. J. Extra Corpor. Technol..

[13] Kalogeris T., Baines C.P., Krenz M., Korthuis R.J. (2016). Ischemia/Reperfusion. Compr. Physiol..

[14] Kalogeris T., Baines C.P., Krenz M., Korthuis R.J. (2012). Cell biology of ischemia/reperfusion injury. Int. Rev. Cell Mol. Biol..

[15] Tsutsui H., Kinugawa S., Matsushima S. (2009). Mitochondrial oxidative stress and dysfunction in myocardial remodelling. Cardiovasc. Res..

[16] Chuah S.C., Moore P.K., Zhu Y.Z. (2007). S-allylcysteine mediates cardioprotection in an acute myocardial infarction rat model via a hydrogen sulfide-mediated pathway. Am. J. Physiol. Heart Circ. Physiol..

[17] Misra M.K., Sarwat M., Bhakuni P., Tuteja R., Tuteja N. (2009). Oxidative stress and ischemic myocardial syndromes. Med. Sci. Monit..

[18] Dai D.F., Chen T., Szeto H., Nieves-Cintron M., Kutyavin V., Santana L.F., Rabinovitch P.S. (2011). Mitochondrial targeted antioxidant Peptide ameliorates hypertensive cardiomyopathy. J. Am. Coll. Cardiol..

[19] Birk A.V., Liu S., Soong Y., Mills W., Singh P., Warren J.D., Seshan S.V., Pardee J.D., Szeto H.H. (2013). The mitochondrial-targeted compound SS-31 re-energizes ischemic mitochondria by interacting with cardiolipin. J. Am. Soc. Nephrol..

[20] Szeto H.H. (2008). Mitochondria-targeted cytoprotective peptides for ischemia-reperfusion injury. Antioxid Redox Signal.

[21] Lu H.I., Huang T.H., Sung P.H., Chen Y.L., Chua S., Chai H.Y., Chung S.Y., Liu C.F., Sun C.K., Chang H.W. (2016). Administration of antioxidant peptide SS-31 attenuates transverse aortic constriction-induced pulmonary arterial hypertension in mice. Acta Pharmacol. Sin..

[22] Zhao K., Zhao G.M., Wu D., Soong Y., Birk A.V., Schiller P.W., Szeto H.H. (2004). Cell-permeable peptide antioxidants targeted to inner mitochondrial membrane inhibit mitochondrial swelling, oxidative cell death, and reperfusion injury. J. Biol. Chem..

[23] Islam M.N., Das S.R., Emin M.T., Wei M., Sun L., Westphalen K., Rowlands D.J., Quadri S.K., Bhattacharya S., Bhattacharya J. (2012). Mitochondrial transfer from bone-marrow-derived stromal cells to pulmonary alveoli protects against acute lung injury. Nat. Med..

[24] Chen H.H., Chen Y.T., Yang C.C., Chen K.H., Sung P.H., Chiang H.J., Chen C.H., Chua S., Chung S.Y., Chen Y.L. (2016). Melatonin pretreatment enhances the therapeutic effects of exogenous mitochondria against hepatic ischemia-reperfusion injury in rats through suppression of mitochondrial permeability transition. J. Pineal Res..

[25] Masuzawa A., Black K.M., Pacak C.A., Ericsson M., Barnett R.J., Drumm C., Seth P., Bloch D.B., Levitsky S., Cowan D.B. (2013). Transplantation of autologously derived mitochondria protects the heart from ischemia-reperfusion injury. Am. J. Physiol. Heart Circ. Physiol..

[26] Lin H.C., Liu S.Y., Lai H.S., Lai I.R. (2013). Isolated mitochondria infusion mitigates ischemia-reperfusion injury of the liver in rats. Shock.

[27] Szeto H.H., Liu S., Soong Y., Wu D., Darrah S.F., Cheng F.Y., Zhao Z., Ganger M., Tow C.Y., Seshan S.V. (2011). Mitochondria-targeted peptide accelerates ATP recovery and reduces ischemic kidney injury. J. Am. Soc. Nephrol..

[28] Kloner R.A., Hale S.L., Dai W., Gorman R.C., Shuto T., Koomalsingh K.J., Gorman J.H., Sloan R.C., Frasier C.R., Watson C.A. (2012). Reduction of ischemia/reperfusion injury with bendavia, a mitochondria-targeting cytoprotective Peptide. J. Am. Heart Assoc..

[29] Dai W., Cheung E., Alleman R.J., Perry J.B., Allen M.E., Brown D.A., Kloner R.A. (2016). Cardioprotective Effects of Mitochondria-Targeted Peptide SBT-20 in two Different Models of Rat Ischemia/Reperfusion. Cardiovasc. Drugs Ther..

[30] Chua S., Lee F.Y., Chiang H.J., Chen K.H., Lu H.I., Chen Y.T., Yang C.C., Lin K.C., Chen Y.L., Kao G.S. (2016). The cardioprotective effect of melatonin and exendin-4 treatment in a rat model of cardiorenal syndrome. J. Pineal Res..

[31] Sheu J.J., Sung P.H., Leu S., Chai H.T., Zhen Y.Y., Chen Y.C., Chua S., Chen Y.L., Tsai T.H., Lee F.Y. (2013). Innate immune response after acute myocardial infarction and pharmacomodulatory action of tacrolimus in reducing infarct size and preserving myocardial integrity. J. Biomed. Sci..

[32] Chen Y.L., Chung S.Y., Chai H.T., Chen C.H., Liu C.F., Chen Y.L., Huang T.H., Zhen Y.Y., Sung P.H., Sun C.K. (2015). Early Administration of Carvedilol Protected against Doxorubicin-Induced Cardiomyopathy. J. Pharmacol. Exp. Ther..

[33] Chen Y.L., Sun C.K., Tsai T.H., Chang L.T., Leu S., Zhen Y.Y., Sheu J.J., Chua S., Yeh K.H., Lu H.I. (2015). Adipose-derived mesenchymal stem cells embedded in platelet-rich fibrin scaffolds promote angiogenesis, preserve heart function, and reduce left ventricular remodeling in rat acute myocardial infarction. Am. J. Transl. Res..

[34] Khalitova E.B. (1974). Viability and retention of virulent properties by shigellae lyophilized in different protective media. Zh. Mikrobiol. Epidemiol. Immunobiol..

[35] Morigi M., Perico L., Rota C., Longaretti L., Conti S., Rottoli D., Novelli R., Remuzzi G., Benigni A. (2015). Sirtuin 3-dependent mitochondrial dynamic improvements protect against acute kidney injury. J. Clin. Investig..

[36] Chua S., Lee F.Y., Tsai T.H., Sheu J.J., Leu S., Sun C.K., Chen Y.L., Chang H.W., Chai H.T., Liu C.F. (2014). Inhibition of dipeptidyl peptidase-IV enzyme activity protects against myocardial ischemia-reperfusion injury in rats. J. Transl. Med..

[37] Huang T.H., Chung S.Y., Chua S., Chai H.T., Sheu J.J., Chen Y.L., Chen C.H., Chang H.W., Tong M.S., Sung P.H. (2016). Effect of early administration of lower dose versus high dose of fresh mitochondria on reducing monocrotaline-induced pulmonary artery hypertension in rat. Am. J. Transl. Res..

[38] Sun C.K., Lee F.Y., Kao Y.H., Chiang H.J., Sung P.H., Tsai T.H., Lin Y.C., Leu S., Wu Y.C., Lu H.I. (2015). Systemic combined melatonin-mitochondria treatment improves acute respiratory distress syndrome in the rat. J. Pineal Res..

[39] Leu S., Sun C.K., Sheu J.J., Chang L.T., Yuen C.M., Yen C.H., Chiang C.H., Ko S.F., Pei S.N., Chua S. (2011). Autologous bone marrow cell implantation attenuates left ventricular remodeling and improves heart function in porcine myocardial infarction: An echocardiographic, six-month angiographic, and molecular-cellular study. Int. J. Cardiol..

